# Porcine Reproductive and Respiratory Syndrome Virus Infection Induces Stress Granule Formation Depending on Protein Kinase R-like Endoplasmic Reticulum Kinase (PERK) in MARC-145 Cells

**DOI:** 10.3389/fcimb.2017.00111

**Published:** 2017-04-04

**Authors:** Yanrong Zhou, Liurong Fang, Dang Wang, Kaimei Cai, Huanchun Chen, Shaobo Xiao

**Affiliations:** ^1^State Key Laboratory of Agricultural Microbiology, College of Veterinary Medicine, Huazhong Agricultural UniversityWuhan, China; ^2^The Cooperative Innovation Center for Sustainable Pig Production, Huazhong Agricultural UniversityWuhan, China

**Keywords:** porcine reproductive and respiratory syndrome virus, stress granule, protein kinase R-like endoplasmic reticulum kinase, replication, inflammatory response

## Abstract

Stress granules (SGs) are sites of mRNA storage that are formed in response to various conditions of stress, including viral infections. Porcine reproductive and respiratory syndrome virus (PRRSV) is an *Arterivirus* that has been devastating the swine industry worldwide since the late 1980s. In this study, we found that infection of PRRSV strain WUH3 (genotype 2 PRRSV) induced stable formation of robust SGs in MARC-145 cells, as demonstrated by the recruitment of marker proteins of SGs, including TIA1, G3BP1, and eIF3η. Treatment with specific inhibitors or siRNAs against the stress kinases that are involved in SG formation revealed that PRRSV induced SG formation through a PERK (protein kinase R–like endoplasmic reticulum kinase)-dependent mechanism. Impairment of SG assembly by concomitant knockdown of the SG marker proteins (TIA1, G3BP1, and TIAR) did not affect PRRSV growth, while significantly enhanced PRRSV-induced NF-κB subunit p65 phosphorylation and inflammatory cytokine production. Taken together, our results demonstrate that PRRSV induces SG formation via a PERK-dependent pathway and that SGs are involved in the signaling pathway of the PRRSV-induced inflammatory response in MARC-145 cells.

## Introduction

Porcine reproductive and respiratory syndrome (PRRS), which is characterized by severe reproductive failure in sows and acute respiratory distress in pigs of all ages, is currently one of the most economically important diseases impacting the swine industry worldwide (Lunney et al., 2010). The causative agent, PRRS virus (PRRSV), is an enveloped, single-stranded positive-sense RNA virus classified within the *Arteriviridae* family (Snijder et al., 2013). Since its emergence in the late 1980s, PRRS has been the most important threat to the global swine industry and is difficult to control through vaccination (Meng, 2000; Neumann et al., 2005). A better understanding of the virus–host interactions will facilitate development of more effective control measures (Loving et al., 2015).

Stress granules (SGs) are discrete cytoplasmic protein–RNA structures that are quickly assembled in eukaryotic cells when the cells are exposed to various stresses, including heat shock, starvation, oxidation, ultraviolet irradiation, hypoxia, and viral infection (Anderson and Kedersha, 2009). Four stress kinases have been identified that are involved in the SG formation when cells are exposed to distinct types of stresses: double-stranded RNA (dsRNA)-dependent protein kinase (PKR) senses dsRNA during viral infection; protein kinase R–like endoplasmic reticulum kinase (PERK) detects unfolded proteins in the endoplasmic reticulum; heme-regulated inhibitor kinase (HRI) monitors changes in heme levels; and general control nonderepressible 2 kinase (GCN2) senses amino acid starvation, ultraviolet damage, and viral infection (Yamasaki and Anderson, 2008). Activation of any of these kinases leads to the phosphorylation of the alpha-subunit of eukaryotic initiation factor 2 (eIF2α), which inhibits the exchange of eIF2-GDP for eIF2-GTP. Thus, phosphorylation of eIF2α decreases the level of the active eIF2/tRNAiMet/GTP ternary complex, leading to the reversible inhibition of translation initiation and consequent polysome disassembly with a corresponding accumulation of the stalled 43S and 48S ribosomal preinitiation complexes (Mazroui et al., 2006). These complexes then recruit multiple RNA-binding proteins (RBPs), including Ras-GTPase-activating protein SH3-domain-binding protein 1 (G3BP1), T-cell internal antigen 1 (TIA1), TIA1-related protein (TIAR), and many others, to nucleate SG assembly (Reineke and Lloyd, 2013). In addition to the most common SG assembly pathway described above, SGs can also be induced by eIF2α-independent mechanisms via the inactivation of translation factors (eIF4A or eIF4G) or the overexpression of SG components, such as TIA1 and G3BP1 (Kedersha et al., 2000; Tourriere et al., 2003). Although many pathways can result in SG or SG-like structure formations, the canonical SGs are defined by the presence of key translation initiation factors, mRNA and the small ribosome subunit (Dinh et al., 2013; Reineke and Lloyd, 2013).

Viral infection can certainly be viewed as a source of stress for cells, and several viruses have been studied to monitor their effect on the host stress response, especially how viruses modulate SG assembly. Many viruses have been reported to inhibit SG accumulation, such as Human T cell leukemia virus type-1 (HTLV-1) (Legros et al., 2011), Rotavirus (Montero et al., 2008), Influenza A virus (IAV) (Khaperskyy et al., 2012), Mengovirus, and Theiler's murine encephalomyelitis virus (TMEV) (Borghese and Michiels, 2011). In contrast, several viruses, such as Respiratory syncytial virus (RSV), induce the stable formation of SGs (Lindquist et al., 2010). Furthermore, transient SG formation is observed during the infection by some viruses, such as Encephalomyocarditis virus (Ng et al., 2013), Poliovirus (White et al., 2007), Coxsackievirus type B3 (CVB3) (Fung et al., 2013), and Mammalian orthoreovirus (MRV) (Qin et al., 2011). Growing evidence suggests that SGs, or at least SG components, may potentially function to repress productive viral infections. The propagation of some viruses, such as Measles virus (Okonski and Samuel, 2013), Japanese encephalitis virus (Katoh et al., 2013), Poliovirus (White et al., 2007), Vesicular stomatitis virus (VSV) (Dinh et al., 2013), Mengovirus, and TMEV (Borghese and Michiels, 2011), can be impaired by SGs or their components. In the case of IAV, mutant IAV that lacks nonstructural protein 1 (NS1), but not wild-type IAV, efficiently generates SGs containing retinoic acid inducible gene I (RIG-I) together with viral RNA and antiviral proteins. Inhibition of these SGs results in an enhancement of IAV replication (Onomoto et al., 2012). Conversely, several viruses have been reported to benefit from SG formation. For example, RSV replication was impaired in cells with a reduced level of G3BP1 expression (Lindquist et al., 2010). Currently, the interplay between PRRSV replication and the cellular stress response remains largely unclear.

In this study, we investigated the relationship between PRRSV and SGs. Our data show that PRRSV induces the stable formation of robust SGs, and this induction depends on the activation of the stress kinase PERK. We also finds that impairment of SG assembly does not affect PRRSV replication, while promotes PRRSV-induced inflammatory cytokines production.

## Materials and methods

### Cell and virus

MARC-145 cells, a monkey kidney cell line highly permissive of PRRSV infection, were cultured and maintained in Dulbecco's modified Eagle's medium (DMEM; Invitrogen, Carlsbad, CA, USA) supplemented with 10% fetal bovine serum, 100 U/mL penicillin, and 100 μg/mL streptomycin sulfate in a humidified 37°C, 5% CO_2_ incubator. PRRSV strain WUH3 (GenBank accession no. HM853673), which was isolated from the brains of pigs suffering from “high fever” syndrome in China at the end of 2006 and identified as a highly pathogenic type 2 (North American) PRRSV, was used throughout this study (Li et al., 2009; Wang et al., 2013). PRRSV was amplified and the resulting titers were assessed in MARC-145 cells. UV-inactivated PRRSV was generated as described previously (Jing et al., 2017). Briefly, About 2 ml of virus suspensions (containing 5.0 × 10^6^ PFU/ml of PRRSV) were dispensed to form a layer of fluid less than 2 mm deep in a round, flat tissue culture plate of 10 cm diameter. The tissue culture plate was placed in clean bench and irradiated within 20 cm far from shortwave (254 nm) ultraviolet light source for 1 h. The loss of infectivity was confirmed by the inability of the UV-irradiated virus to produce a cytopathic effect on monolayer of MARC-145 cells.

### Reagents, siRNAs, and cell transfection

PERK-I (516535 PERK Inhibitor I, GSK2606414; solubilized in dimethyl sulfoxide (DMSO)) and an imidazolo-oxindole compound (C16) were purchased from Merck Millipore (Darmstadt, Germany) and were used at concentrations of 1 and 0.5 μM, respectively. Sodium arsenite (SA) and 2-aminopurine (2-AP; dissolved in DMSO) were obtained from Sigma-Aldrich and (St Louis, MO, USA) were used at concentrations of 500 μM and 0.25 mM, respectively. The siRNAs targeting monkey TIA1, G3BP1, HRI, or GCN2 (catalog no. 10620312, Invitrogen) or negative control siRNA (catalog no. 12935; Invitrogen) were each transfected at a final concentration of 50 nM using Lipofectamine 2000 reagent (Invitrogen) according to the manufacturer's instructions. The siRNA sequences used in these protocols are listed in Table 1.

**Table 1:** **The sequences of siRNAs used in this study**.

**Gene name**	**siRNA sequence (sense 5′–3′)**	**siRNA sequence (anti-sense 5′–3′)**
TIA1	AAGAGUUGCAGAAUUAGAGCUUCUG	CAGAAGCUCUAAUUCUGCAACUCUU
G3BP1	UCAACAUGGCGAAUCUUGGUGUGGC	GCCACACCAAGAUUCGCCAUGUUGA
TIAR	GGUAGUUAAAGACAUGGCAACUGGA	UCCAGUUGCCAUGUCUUUAACUACC
HRI	UUUAGUUGCACCCUUAAUCAGGAUC	GAUCCUGAUUAAGGGUGCAACUAAA
GCN2	CAAGAUGCAGCAGUAUGUAUGUGAA	UUCACAUACAUACUGCUGCAUCUUG

### Antibodies

The rabbit monoclonal antibodies (mAb) against phospho-eIF2α (Ser51) and eIF2α were purchased from Cell Signaling Technology (Beverly, MA, USA). Mouse mAb against beta-actin was obtained from Medical and Biological Laboratories (MBL, Nagova, Japan). Antibodies against TIA1 (goat polyclonal), G3BP1 (rabbit polyclonal), TIAR (goat polyclonal), and eIF3η (goat polyclonal) were purchased from Santa Cruz Biotechnology, Inc (Santa Cruz, CA, USA). Rabbit mAb against NF-κB p65 and phospho-NF-κB p65 were purchased from Cell Signaling Technology. Mouse mAb against PRRSV nucleocapsid (N) protein was prepared by our laboratory. Alexa Fluor 594-conjugated donkey anti-goat IgG, Alexa Fluor 594-conjugated donkey anti-rabbit IgG, Alexa Fluor 488-conjugated donkey anti-rabbit IgG, Alexa Fluor 488-conjugated donkey anti-mouse IgG, and Alexa Fluor 647-conjugated donkey anti-mouse IgG were also obtained from Santa Cruz Biotechnology, Inc. Horseradish peroxidase (HRP)-conjugated goat anti-rabbit, HRP-conjugated goat anti-mouse, and HRP-conjugated donkey anti-goat were purchased from Medical and Biological Laboratories (MBL).

### Indirect immunofluorescence assay

MARC-145 cells were seeded on circular glass coverslips in 24-well plates and left to grow until they reached 80–90% confluence. At the indicated time points after treatment, the cells were fixed with 4% paraformaldehyde for 15 min and immediately permeabilized with methanol that had been pre-cooled at −20°C for 10 min. The fixed and permeabilized cells were blocked with 5% bovine serum albumin (BSA) in phosphate-buffered saline (PBS) for 45 min, followed by incubation with primary antibodies for 1 h. The cells were then incubated with Alexa Fluor-labeled secondary antibodies for 1 h, after which they were counterstained with 4′,6-diamidino-2-phenylindole (DAPI; Beyotime, Nantong, China) in PBS (1/200 dilution) for 15 min. The resulting fluorescent images were acquired with an Olympus FV10 laser scanning confocal microscope (Olympus, Japan).

### Western blot analysis

Cell lysates were obtained by the addition of lysis buffer (4% sodium dodecyl sulfate (SDS), 3% dithiothreitol (DTT), 0.065 mM Tris-HCl, [pH 6.8], and 30% glycerine) supplemented with protease inhibitor (PMSF). Equal amounts of samples were subjected to sodium dodecyl sulfate polyacrylamide gel electrophoresis (SDS-PAGE), and the separated proteins were electroblotted onto a polyvinylidene difluoride (PVDF) membrane (Millipore, Billerica, MA, USA). The expressions of TIA1, G3BP1, eIF2α, or phospho-eIF2α protein were analyzed using appropriate specific antibodies. Specific mAb against PRRSV N (diluted 1:1,000) was used to analyze the expression of N protein. Beta-actin was detected with an anti-beta-actin mouse mAb (Beyotime, Nantong, China) as a loading control.

### Plaque assay for determination of PRRSV titers

Plaque assays were conducted essentially as described previously (Luo et al., 2011). Briefly, 95% confluent MARC-145 cells grown in six-well tissue culture plates were infected for 1 h with 10-fold serial dilutions (1 ml each) of PRRSV-containing samples. After three washes with PBS (pH 7.4), the cells were overlaid with 1.8% (w/v) Bacto agar mixed 1:1 with 2 × DMEM containing 0.05 mg/ml neutral red. Plaques were counted 2 days post-infection. The average plaque numbers and the standard deviations were calculated from three independent experiments. Virus titers are expressed as PFU/mL.

### Quantitative real-time PCR (RT-qPCR)

Total RNA was extracted from the treated cells using TRIzol reagent (Invitrogen) according to the manufacturer's instructions, and 1 μg of each sample was subsequently reverse transcribed to cDNA by using oligo-dT primer with a Transcriptor First Strand cDNA Synthesis Kit (Roche, Mannheim, Germany) according to the manufacturer's instructions. The resulting cDNA (1 μl of the 20-μl RT reaction mixture) was then used as the template in a SYBR green qPCR assay (Applied Biosystems, Foster City, CA, USA). The abundance of individual mRNA transcripts in each sample was assayed three times and normalized to that of glyceraldehyde-3-phosphate dehydrogenase (GAPDH) mRNA (the internal control). Gene expression was measured as described previously (Wang et al., 2012). The primers (Table 2) were designed with Primer Express software v.3.0 (Applied Biosystems).

**Table 2:** **Primers used in the quantitative real-time PCR**.

**Primer names**	**Sequence (5′–3′)**
mGAPDH-qF	ACATGGCCTCCAAGGAGTAAGA
mGAPDH-qR	GATCGAGTTGGGGCTGTGACT
mTIA1-qF	CCATGGATGGGACCAAATTA
mTIA1-qR	TTTCATACCCTGCCACTCGAT
mG3BP1-qF	GGGCGGGAATTTGTGAGA
mG3BP1-qR	ATCCCCCATGGACATAAGAAG
mTIAR-qF	GGCAACCATGGAATCAACAA
mTIAR-qR	CCAGCTTGGTTAGGAGGAGGTA
mHRI-qF	CGGGAAGAGAACACCAACAC
mHRI-qR	AGGATCACACCCAAGCTGTAC
mGCN2-qF	TCCAGTTTGTGGCATTCATC
mGCN2-qR	CAGAGCTTGTGGCCCTCTAA
mIL-6-qF	GCTGCAGGCACAGAACCA
mIL-6-qR	AAAGCTGCGCAGGATGAGA
mIL-8-qF	CTGGCGGTGGCTCTCTTG
mIL-8-qR	CCTTGGCAAAACTGCACCTT
mTNF-α-qF	TCCTCAGCCTCTTCTCCTTCCT
mTNF-α-qR	ACTCCAAAGTGCAGCAGACAGA

### Induction and quantification of SGs

MARC-145 cells were infected with PRRSV at a multiplicity of infection (MOI) of 1 for 0, 6, 12, 24, 36, or 48 h. Cells were fixed as described above. As a positive control, cells were treated with SA for 1 h to induce SGs. At least 10 high-powered fields (HPFs) were randomly obtained with at least 250 cells counted for each sample. The total number of cells was quantified by counting the number of nuclei. PRRSV N was used to monitor PRRSV infection. We also counted the number of SG-positive cells per HPF as assessed by the results of staining for the presence of G3BP1, TIA1, or eIF3η proteins.

### Statistical analysis

The one-way analysis of variance (ANOVA) was applied to analyze the experimental data. Data of three independent experiments were shown as the mean ± standard deviation (SD). Statistical significance was determined with a *p*-value < 0.01.

### Ethics statement

All animal experiments were approved by the Hubei Administrative Committee for Laboratory Animals (permission number 00024534) and complied with the guidelines of Hubei laboratory animal welfare and ethics of Hubei Administrative Committee of Laboratory Animals.

## Results

### PRRSV infection induces stable SG formation depending on viral replication

**Figure 1:**
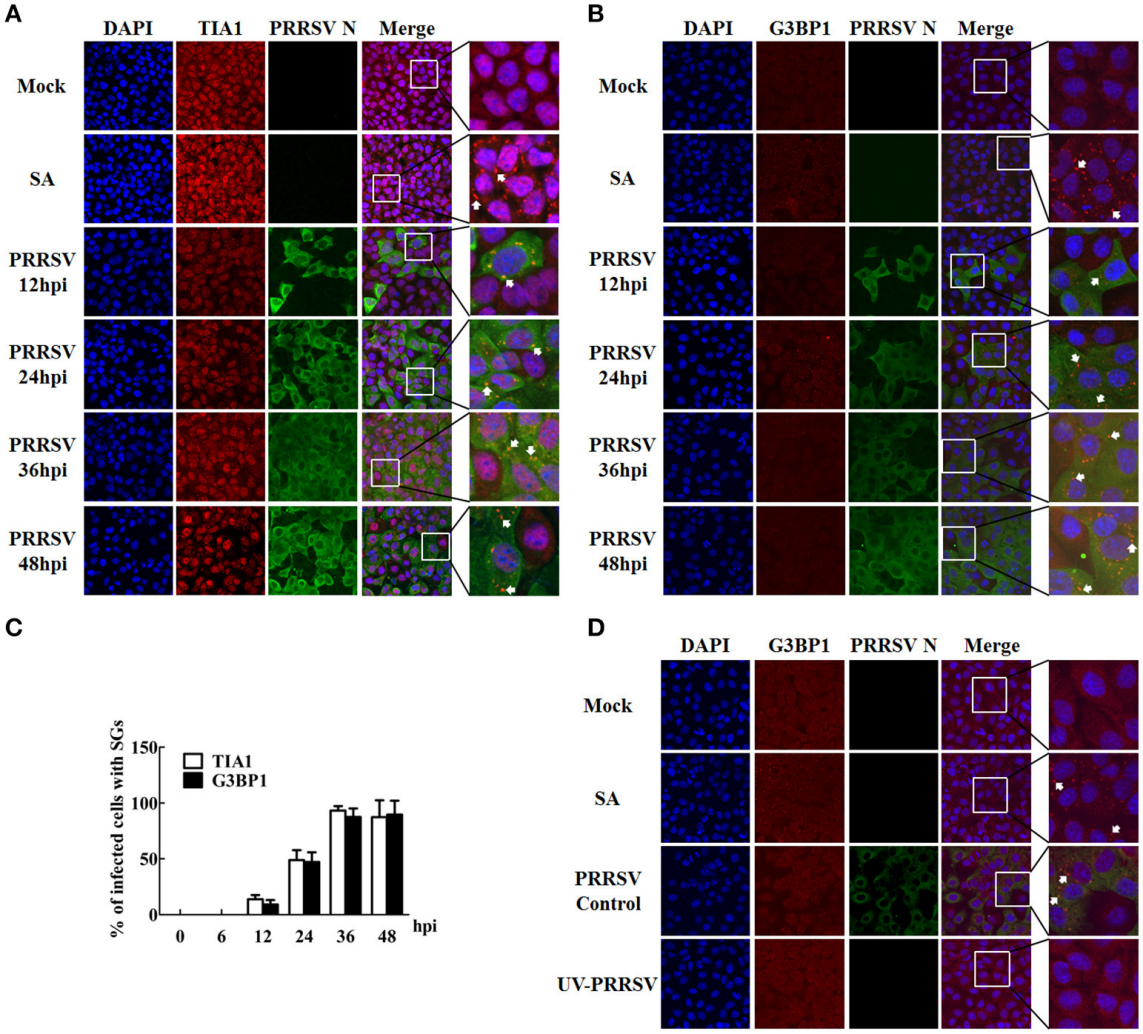
**PRRSV infection induces the formation of stress granules. (A,B)** MARC-145 cells were mock-infected or infected with PRRSV for the indicated times. As a positive control, uninfected cells were treated with 0.5 mM (sodium arsenite, SA) for 1 h. Cells were fixed and analyzed by confocal microscopy. Goat polyclonal antibody specific for TIA1 (**A**; red) or rabbit polyclonal antibody specific for G3BP1 (**B**; red) was used to detect SGs. Mouse monoclonal antibody specific for PRRSV N was used to detect PRRSV-infected cells (green). Nuclei were stained with DAPI (blue). The arrows point the SGs in cytoplasm. **(C)** The total number of PRRSV-infected cells and the number of PRRSV-infected cells containing SGs were counted, and the percentage of SG-positive PRRSV-infected cells per HPF were quantified at various time points after infection as described in the Materials and Methods. Error bars show standard deviations. **(D)** MARC-145 cells were mock-inoculated or inoculated with replication-competent PRRSV (PRRSV control), or an equivalent volume of UV-inactivated PRRSV (UV-PRRSV) for 36 h. The cells were then analyzed for G3BP1 (red) and PRRSV N (green) expression as described for panel **(B)**. Nuclei were stained with DAPI (blue). The arrows point the SGs in cytoplasm.

To investigate whether or not PRRSV infection induces SGs, MARC-145 cells were infected with PRRSV strain WUH3 and the localizations of endogenously expressed SG marker proteins (G3BP1 and TIA1) were examined by performing indirect immunofluorescence assays. Because the formation of SGs is kinetic, five time points (6, 12, 24, 36, and 48 h) after PRRSV infection were examined. Cells treated with the potent SG-inducer, sodium arsenite (SA), and mock-infected cells served as positive and negative controls, respectively. Monoclonal antibody specific for PRRSV N was used to detect PRRSV-infected cells. As expected, more than 95% of SA-treated cells contained SGs, while no obvious SGs were observed in mock-infected cells, which is consistent with findings from a previous report (McEwen et al., 2005). In PRRSV-infected cells, cytoplasmic granules containing SG marker proteins TIA1 or G3BP1 were not seen at 6 h post-infection (hpi) (data not shown), but they began to occur at 12 hpi and still existed at 48 hpi (Figures 1A,B). Based on the number of PRRSV-infected cells containing SGs, PRRSV triggered the formation of SGs in approximately 10% of cells at 12 hpi, 50% of cells at 24 hpi, and 90% of cells at 36 and 48 hpi (Figure 1C). These results suggest that a robust SG response was induced by PRRSV beginning at approximately 12 h after inoculation and maintained during the course of PRRSV infection.

To examine whether or not PRRSV replication is necessary for SG formation, we examined the ability of UV-inactivated PRRSV to induce SGs. To this end, MARC-145 cells were inoculated or mock-inoculated with UV-inactivated PRRSV for 36 h and then fixed and stained with antibodies against G3BP1 and PRRSV N. Cells infected with PRRSV were used as positive controls. As shown in Figure 1D (second and third rows), both SA treatment and PRRSV infection induced robust SG formation, while SG formation was not detectable in cells that were mock-inoculated or inoculated with UV-inactivated PRRSV (Figure 1D, top and bottom rows, respectively). These results indicate that PRRSV replication is required for SG formation.

### PRRSV-induced SGs are similar to canonical SGs

**Figure 2:**
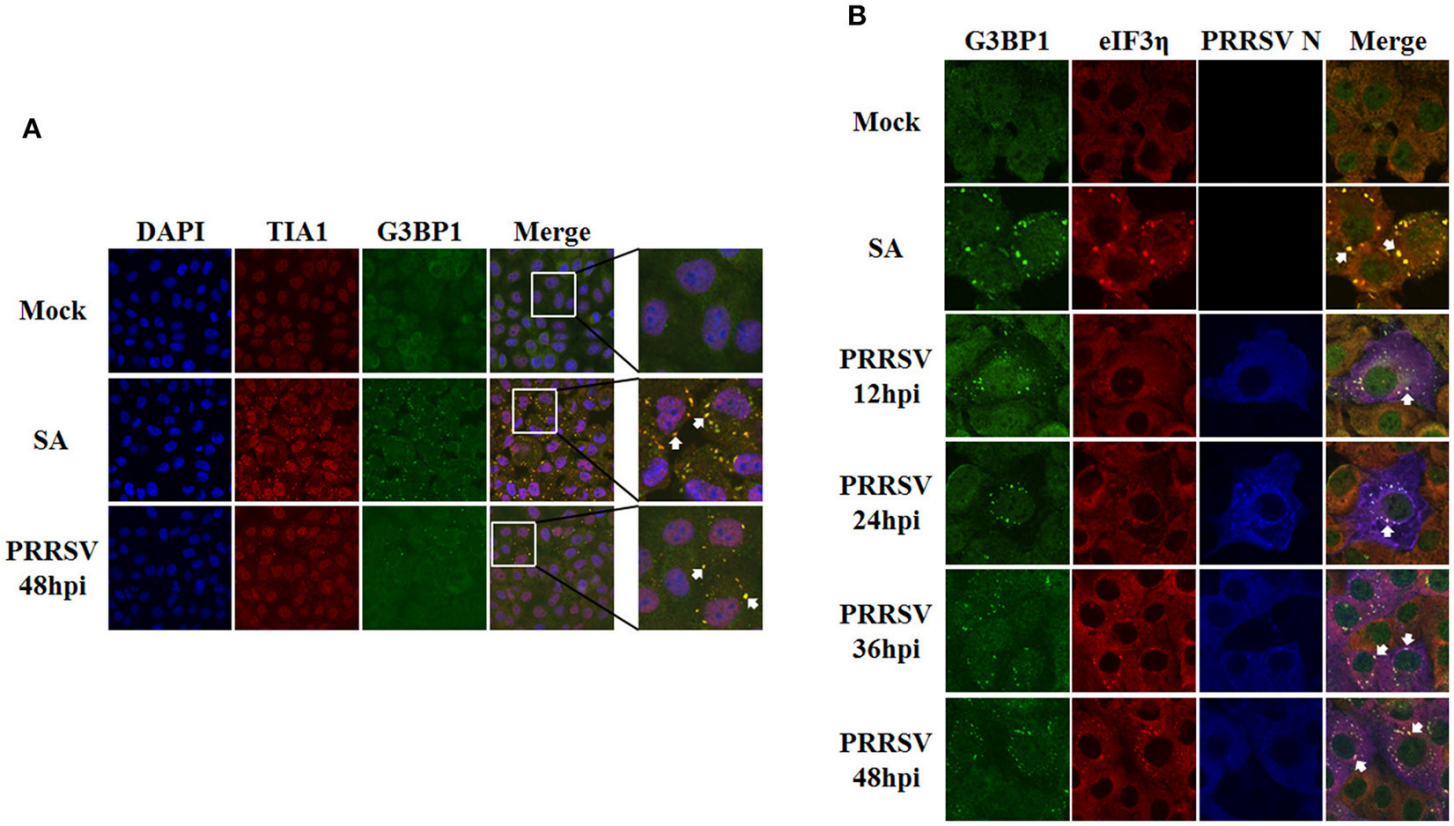
**Comparison between SGs triggered by PRRSV and those induced by SA. (A)** Localization of G3BP1 (green) and TIA1 (red) in PRRSV-infected cells. MARC-145 cells were mock-infected or infected with PRRSV. As a positive control, uninfected cells were treated with 0.5 mM SA for 1 h. At 36 hpi, the cells were fixed, stained with anti-G3BP1 antibody and anti-TIA1 antibody, followed by staining with Alexa 594-conjugated donkey anti-goat IgG for TIA1 and Alexa 488-conjugated donkey anti-rabbit IgG for G3BP1. Nuclei were stained with DAPI (blue). Stained cells were examined by confocal microscope. The arrows point the SGs in cytoplasm. **(B)** MARC-145 cells were mock-infected or infected with PRRSV. As a positive control, cells were treated with SA as described for **(A)**. At the indicated times, the cells were fixed and analyzed by confocal microscopy. Rabbit polyclonal antibody specific for G3BP1 (green) was used to detect SGs induced by PRRSV, goat polyclonal antibody specific for eIF3η (red) was used to detect SGs triggered by SA, and mouse monoclonal antibody specific for PRRSV N (blue) was used to detect PRRSV-infected cells. The arrows point the SGs in cytoplasm.

Previous studies showed that Vesicular stomatitis virus (VSV) induces SG-like structures that contain the SG marker TIA1 but do not contain some of the bona fide SG markers, such as eukaryotic initiation factor 3 (eIF3) or eIF4A (Dinh et al., 2013). To gain insight into the composition of PRRSV-induced SGs and to determine whether PRRSV-induced SGs are canonical SGs or not, MARC-145 cells were infected with PRRSV strain WUH3 for 36 h, and then TIA1 and G3BP1 expressions were examined by immunostaining. We found that G3BP1 and TIA1 co-localized to PRRSV-induced foci that were similar to the canonical SGs induced by SA (Figure 2A). Because both TIA1 and G3BP1 granules appeared by 12 hpi and remained thereafter (Figures 1A,B), G3BP1 maybe co-localize with TIA1 within SGs throughout PRRSV infection.

To further confirm that the PRRSV-induced SGs are canonical SGs, MARC-145 cells were infected with PRRSV and the cells were fixed for three-color immunofluorescence assays at 12, 24, 36, or 48 hpi. Considering that (i) canonical SGs contain translation initiation factors (such as eIF3η) and (ii) the results above revealed that G3BP1 was recruited to the PRRSV-SGs, goat antibodies against eIF3η were used to visualize the canonical SGs, rabbit antibodies against G3BP1 were used to visualize PRRSV-SGs, and mouse antibodies against PRRSV N protein were used to visualize the infected cells. We found that, early in infection (12 and 24 hpi), hardly any eIF3η foci were observed (Figure 2B, the third and fourth rows, respectively). Interestingly, at later times in infection (36 and 48 hpi), the amount of eIF3η foci was almost as high as that of G3BP1 foci, and both proteins were recruited to the same granules in the PRRSV-infected cells (Figure 2B, the fifth and bottom rows, respectively), which is similar to SA-induced SGs (Figure 2B, the second row). Taken together, these results indicate that the composition of PRRSV-induced SGs is the same as that of canonical SGs. However, the reason why eIF3 is recruited to PRRSV-induced SGs late in infection rather than early, like G3BP1, remains a question for further study.

### Formation of SGs induced by PRRSV is dependent on PERK

**Figure 3:**
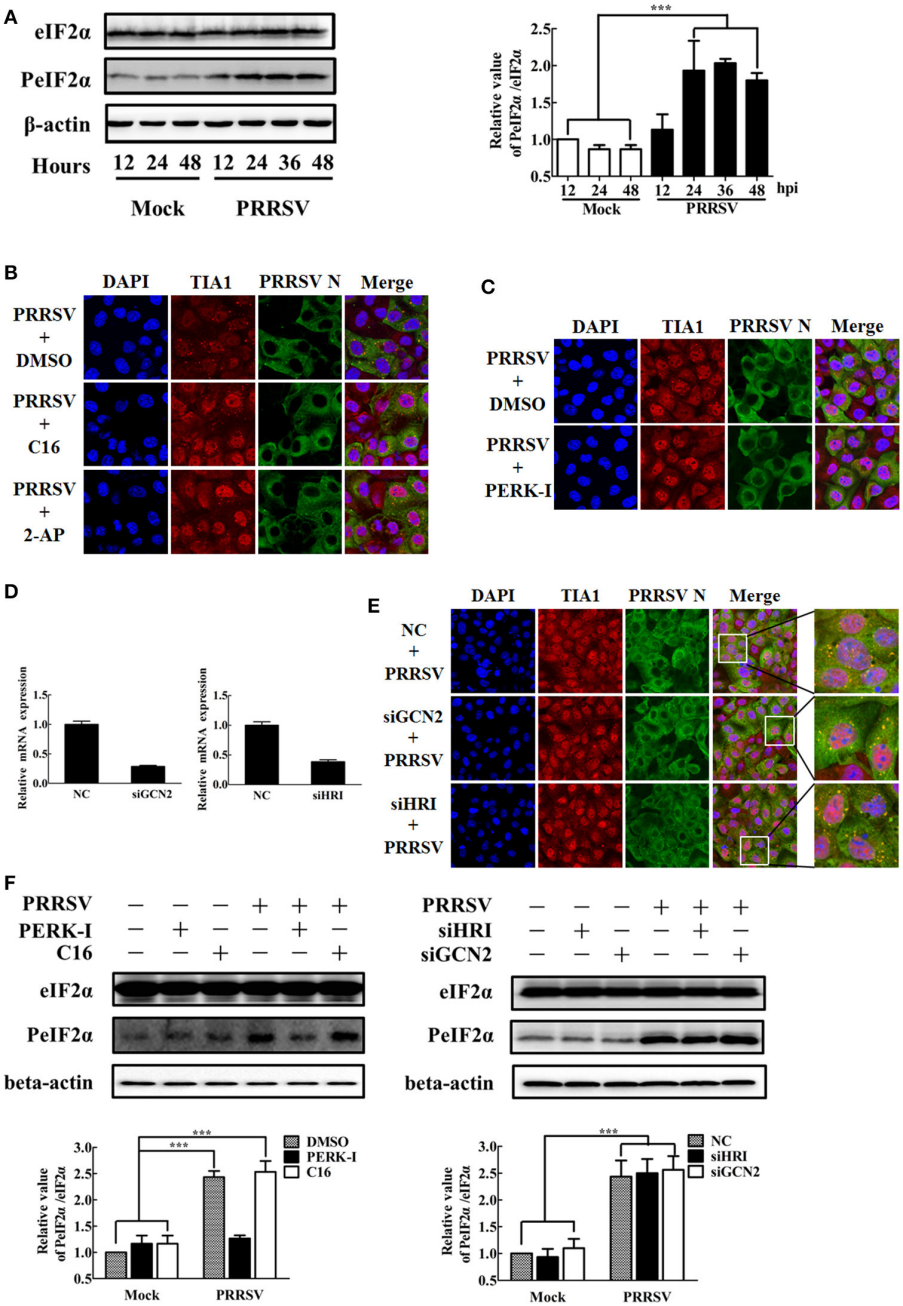
**The SGs induced by PRRSV is PERK dependent. (A)** MARC-145 cells were mock-infected (Mock) or infected with PRRSV (PRRSV). Cell lysates were prepared at the indicated times post-infection, and the eIF2α and phosphorylated eIF2α expression levels were analyzed by Western blotting, using β-actin expression as a control. The representative Western blot was shown and the ratio of phosphorylated/total eIF2α from three independent experiments was analyzed using ImageJ Software. ^***^P < 0.01, comparison between mock-infected and PRRSV-infected cells at indicated times. **(B)** MARC-145 cells were pretreated with PKR inhibitors, 2-AP (0.25 mM) or C16 (0.5 μM), or with vehicle alone (DMSO) for 1 h each. Cells then were inoculated with PRRSV for 1 h. The inoculum was subsequently removed and replaced with medium containing the same concentration of 2-AP, C16, or vehicle as described above. The cells were fixed at 36 hpi and analyzed for TIA1 (red) and PRRSV N (green) expression as described for Figure 1A to assess SG assembly. Nuclei were stained with DAPI (blue). **(C)** MARC-145 cells were pretreated with 1 μM of PERK inhibitor (PERK-I) or vehicle (DMSO) for 1 h each. The cells were then inoculated with PRRSV for 1 h. The inoculum was removed and replaced with medium containing the same concentration of PERK-I or vehicle as described above. Cells then were treated as described for Figure 1A. **(D)** Assessment of the silencing efficiency of GCN2- or HRI-specific siRNAs. MARC-145 cells were transfected with 50 nM/well of siGCN2, siHRI, or negative control (NC) for 36 h each. The resulting mRNA levels of GCN2 or HRI were determined by RT-qPCR. **(E)** MARC-145 cells were transfected with siGCN2, siHRI, or NC for 24 h and then infected with PRRSV for 36 h. Cells were subsequently fixed and analyzed for TIA1 (red) and PRRSV N (green) as described for Figure 1A. **(F)** MARC-145 cells mock-infected or infected with PRRSV were treated with a PKR inhibitor (C16) or a PERK inhibitor (PERK inhibitor) (left) or with siGCN2, siHRI, or NC (right) as described above. Cell lysates were prepared at the 36 hpi, and the eIF2α and phosphorylated eIF2α expression levels were analyzed by Western blotting using β-actin expression as a loading control. The representative Western blot was shown and the ratio of phosphorylated/total eIF2α from three independent experiments was analyzed using ImageJ Software. ^***^P < 0.01, comparison between mock-infected and PRRSV-infected cells at indicated times.

It is well known that the four identified stress kinases, PKR, PERK, GCN2, and HRI, are involved in SG assembly through phosphorylating eIF2α (Sonenberg and Hinnebusch, 2009). We thus analyzed the eIF2α phosphorylation level of MARC-145 cells at different time points after PRRSV infection. As shown in Figure 3A, the increase in eIF2α phosphorylation began by 12 h post-inoculation, which was consistent with previous research (Huo et al., 2013), and maintained throughout infection, and this timing is consistent with the appearance of SGs. These results prompted us to investigate the pathway underlying PRRSV-induced formation of SGs.

To assess the contribution of each individual stress kinase to SG formation following PRRSV infection, we treated MARC-145 cells with specific inhibitors or siRNAs against each of the four stress kinases. The C16 compound and the nucleotide analog 2-AP are potent inhibitors of PKR activity (Hu and Conway, 1993; Jammi et al., 2003); GSK2606414 (henceforth referred to as PERK-I) acts as a highly potent PERK inhibitor by targeting PERK in its inactive DFG conformation at the ATP-binding region (Harding et al., 2012). No appreciable cytotoxicity in MARC-145 cells was detected at concentrations of 0.25–1 μM for C16, 0.25–1 mM for 2-AP, or 0.5–5 μM for PERK-I, as demonstrated by the results of MTT assays (data not shown). To test the effect of these inhibitors on PRRSV-induced SG formation, MARC-145 cells were treated with 2-AP, C16, PERK-I, or vehicle for 1 h followed by PRRSV infection for 1 h. The inoculum was then removed, and the cells subsequently were maintained for 36 h in medium with each individual stress kinase inhibitor or vehicle. The cells were then fixed and stained with antibodies against TIA1 and PRRSV N to examine the formation of SGs. As shown in Figure 3B, an equivalent frequency of SG formation was induced after PRRSV infection in cells treated with 2-AP, C16, or vehicle. However, PRRSV-induced SG formation was substantially reduced in cells treated with PERK-I (Figure 3C, bottom row) compared with that in cells treated with DMSO. These results suggest that PERK rather than PKR is required for SG formation in response to PRRSV infection.

Because no GCN2- or HRI-specific inhibitors were available, specific siRNAs were used to test the effects of GCN2 and HRI on PRRSV-induced SG formation. The designed GCN2- or HRI-specific siRNAs used in this study were able to reduce the endogenous mRNA levels of GCN2 or HRI in MARC-145 cells by over 70 or 60%, respectively, as compared with the negative control (NC) siRNA (Figure 3D). MARC-145 cells were transfected with GCN2- or HRI-specific siRNAs or NC siRNA prior to PRRSV infection. These cells were fixed and immunostained at 36 hpi to examine the presence of SGs. We did not find any significant changes in PRRSV-induced SG formation between either the GCN2-knockdown cells or the HRI-knockdown cells and the NC-treated cells, indicating that neither the depletion of GCN2 nor the depletion of HRI affects the formation of PRRSV-induced SGs (Figure 3E).

To further confirm the pathway of SG induction by PRRSV infection, we measured the relative levels of phosphorylated eIF2α in MARC-145 cells treated with specific inhibitors or siRNAs targeting the four stress kinases as described above. Cells were harvested for Western blot analyses to assess the levels of total or phosphorylated eIF2α. As show in Figure 3F, infection with PRRSV resulted in a strong increase in the eIF2α phosphorylation level, which was diminished in cells treated with PERK-I, but not in cells treated with any of the other specific inhibitors or siRNAs. As compared with treated cells, no similar changes in the levels of phosphorylated eIF2α were observed in the absence of PRRSV infection. Additionally, there was no significant difference in the total eIF2α levels among the various treatments. These results support the conclusion that PRRSV triggers SG assembly via eIF2α phosphorylation, which is dependent on PERK.

### SG disassembly has no prominent influence on PRRSV replication

**Figure 4:**
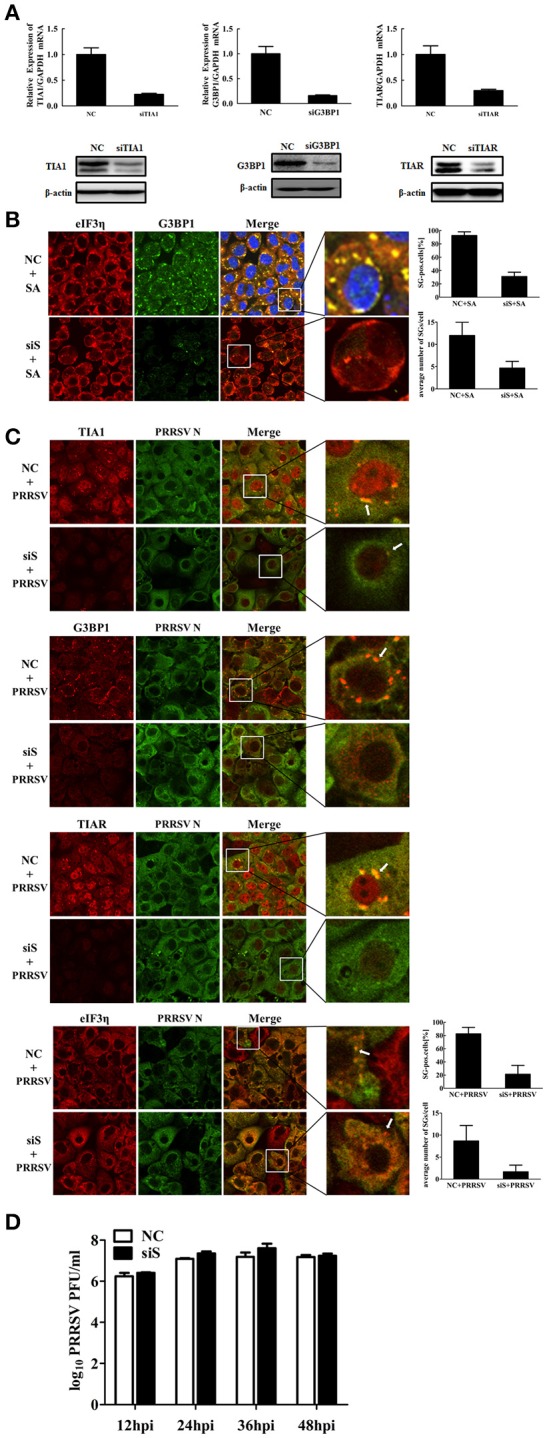
**Effects of SGs disassembly on PRRSV growth. (A)** The silencing efficiency of TIA1-, G3BP1-, or TIAR- specific siRNAs. MARC-145 cells were transfected with 50 nM/well siTIA1, siG3BP1, siTIAR or negative control (NC) siRNA for 36 h each. The expression levels of TIA1 (left), G3BP1 (middle), or TIAR (right) were determined by RT-qPCR (top) and Western blotting (bottom). **(B)** MARC-145 cells were transfected with NC siRNA or siS, the siRNA pools of TIA1, TIAR, and G3BP1, then treated with 0.5 mM SA for 1 h, stained with antibodies against G3BP1 or eIF3η, followed by staining with Alexa 594-conjugated donkey anti-goat IgG for eIF3η, Alexa 488-conjugated donkey anti-rabbit IgG for G3BP1. Nuclei were stained with DAPI (blue). Stained cells were examined by confocal microscope. The percentage of SG-positive cells (up) and average number of SGs per cell (down) were quantified. Error bars show standard deviations. **(C)** MARC-145 cells were simultaneously transfected with siTIA1, siG3BP1 and siTIAR for 24 h, and then infected with PRRSV. At 36 hpi, the cells were fixed, stained with antibodies against PRRSV N, TIA1, G3BP1, TIAR, or eIF3η, followed by staining with Alexa 594-conjugated donkey anti-goat IgG for TIA1, TIAR, or eIF3η, Alexa 594-conjugated donkey anti-rabbit IgG for G3BP1, Alexa 488-conjugated donkey anti-mouse IgG for PRRSV N. Nuclei were stained with DAPI (blue). The stained cells were examined by confocal microscope. The percentage of SG-positive PRRSV-infected cells (up) and average number of SGs per cell (down) were quantified. Error bars show standard deviations. The arrows point the SGs in cytoplasm. **(D)** MARC-145 cells are treated as described in **(C)**. Then the cells and their supernatants were collected together at the indicated times post-infection to determine their viral titers by plaque assay.

A growing body of evidence suggests that the virus-induced SGs or SG components play important roles in the replication of some viruses (Lindquist et al., 2010; Onomoto et al., 2012; Katoh et al., 2013; Reineke and Lloyd, 2015). To assess whether SGs is associated with PRRSV growth, we tried to impair SG formation through concomitant knockdown of TIA1, G3BP1, and TIAR. Firstly, specific siRNAs were designed to knock down the endogenous TIA1, G3BP1, and TIAR, respectively. The knockdown efficiency was demonstrated by the results of real-time RT-qPCR and Western blot assays (Figure 4A). The specific siRNAs targeting TIA1, G3BP1 or TIAR were transfected into MARC-145 cells concomitantly, followed by PRRSV infection or SA treatment. Then SG assembly was monitored upon immunostaining of SG component eIF3η. In line with previous study (Bley et al., 2015), the concomitant depletion of all three proteins by siS, led to substantial SG disassembly in cells treated with SA (Figure 4B). Similar results could be observed in PRRSV-infected cells, indicating that SG assembly induced by PRRSV was aborted sharply when all three proteins, TIA1, G3BP1 and TIAR, were silenced together (Figure 4C, the bottom row). Besides, these cells were also fixed and stained with antibodies against TIA1, G3BP1, and TIAR, respectively, to confirm the efficient knockdown of each protein using indirect immunofluorescence assay (Figure 4C). Then cells were transfected concomitantly with TIA1-, TIAR-, and G3BP1-specific siRNAs, prior to PRRSV infection, and harvested together with the supernatants at 12, 24, 36, or 48 hpi, and plaque assays were performed to assess their titers. Unexpectedly, we found that simultaneous depletion of TIA1, G3BP1, or TIAR caused no significant difference in PRRSV replication compared with control levels (Figure 4D). These results indicate that SGs have no prominent influence on PRRSV replication.

**Figure 5:**
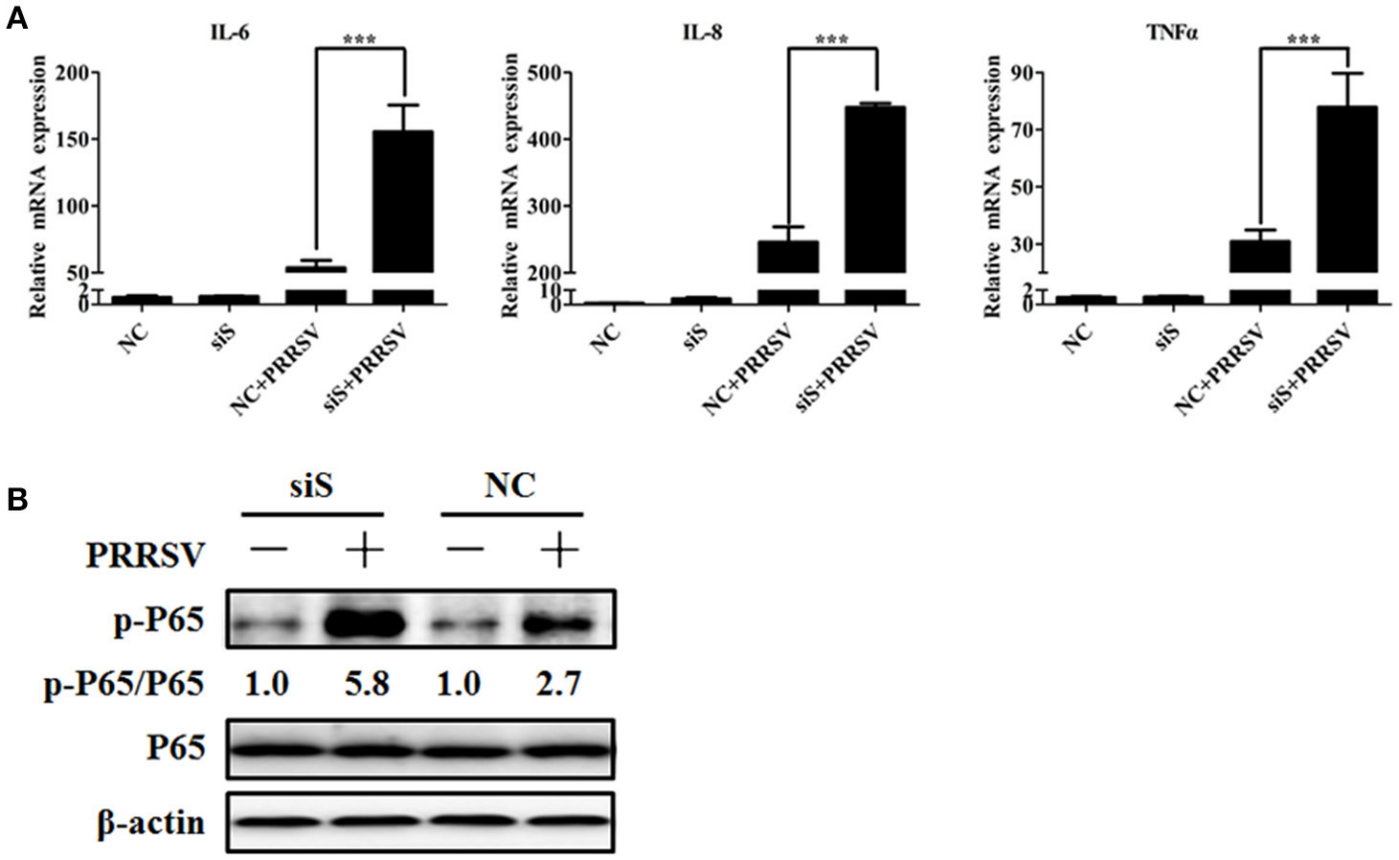
**Role of SGs in the PRRSV-induced inflammatory responses. (A)** MARC-145 cells were simultaneously transfected with siTIA1, siG3BP1, and siTIAR for 24 h, and cells were then mock-infected or infected with PRRSV. Cytokine mRNA levels (IL-6, IL-8, and TNF-α) were evaluated using real-time RT-qPCR assays at 36 hpi. ^***^P < 0.01, comparison between cells transfected with NC siRNA and specific siRNA followed by PRRSV infection. **(B)** MARC-145 cells are treated as described for **(A)**. At 36 hpi, the levels of p65 phosphorylation were evaluated by Western blotting. The ratio of phosphorylated/total p65 was analyzed using ImageJ Software.

The PRRSV-induced inflammatory response is increased in the absence of SGs Previous studies have shown that Roquin, another SG component, affects the expression of interleukin (IL)-6 and tumor necrosis factor (TNF) (Athanasopoulos et al., 2010; Mino et al., 2015). Interstitial pneumonia is considered one of the most remarkable features of PRRSV infection (Duan et al., 2014), and a large number of studies have reported that PRRSV infection induces inflammatory cytokine production *in vivo* and *in vitro* (Gómez-Laguna et al., 2010; Fu et al., 2012). To investigate if SGs have a biological impact on PRRSV-induced pro-inflammatory cytokines, MARC-145 cells were transfected simultaneously with TIA1-, TIAR-, and G3BP1-specific siRNAs prior to PRRSV infection. Then the mRNA expression of some pro-inflammatory cytokines was detected by real-time RT-qPCR. As shown in Figure 5A, the IL-6, IL-8, and TNF-α mRNA expression levels in the concomitant depletion cells were increased to varying degrees compared with those for the negative control-transfected cells.

Because NF-κB is an important transcription factor for pro-inflammatory cytokine production (Tak and Firestein, 2001) and PRRSV infection activates the NF-κB pathway (Fang et al., 2012), we examined if SGs participate in PRRSV-induced NF-κB activation. NF-κB subunit p65 phosphorylation results in NF-κB activation, so we further examined the phosphorylation level of the p65 subunit. As show in Figure 5B, the PRRSV-induced p65 phosphorylation level increased in the cells transfected with siRNA pools of TIA1-, TIAR-, and G3BP1. Notably, the total p65 level remained unchanged in cells transfected with the specific siRNAs compared with those for the negative control-transfected cells.

## Discussion

Our knowledge about SGs has rapidly advanced in recent years. At least four distinct patterns of SG formation have been reported following infection with RNA or DNA viruses: stable, transient, oscillating, or no SG formation (Onomoto et al., 2014). For example, RSV triggers stable SG formation throughout the viral life cycle (Lindquist et al., 2010, 2011), while poliovirus infection causes a transient SG formation during the early phase of infection, but disperses the resulting SGs by cleavage of G3BP1 during later stages of infection (White et al., 2007). The levels of growth arrest DNA-damage-inducible 34 (GADD34) and the phosphorylated form of eIF2α contribute to the oscillating SG formation in hepatitis C virus-infected cells (Ruggieri et al., 2012). In contrast, Mengovirus, TMEV (Borghese and Michiels, 2011), Herpes simplex virus (Khong and Jan, 2011), and Influenza virus (Khaperskyy et al., 2012) all restrict SG accumulation. In our present study, we found that PRRSV induces SG formation within 12 h of inoculation and that the frequency of SGs increases as the infection progresses, indicating that PRRSV specifically initiates and maintains the stress response in MARC-145 cells. Previous studies showed that the infection of transmissible gastroenteritis coronavirus (TGEV) and mouse hepatitis coronavirus (MHV), both viruses belong to *Nidovirales* order as PRRSV, could also induce SG formation (Raaben et al., 2007; Sola et al., 2011). Whether it is a common feature of *Nidovirales* viruses to induce SGs requires future works.

Four mammalian serine-threonine kinases targeting eIF2α (PKR, HRI, GCN2, and PERK) are involved in the stress responses to various environments. Abundant evidence indicates that several viruses commonly induce SG formation via the activation of PKR, or in some cases, via the activation of GCN2 (Berlanga et al., 2006; Lindquist et al., 2011; Okonski and Samuel, 2013), while other viruses block SG formation by preventing PKR activation, such as IAV (Khaperskyy et al., 2012) and West Nile virus (Courtney et al., 2012). Alternately, some viruses induce SG assembly, but the formation of SGs is abolished by PKR deficiency, such as hepatitis C virus (Garaigorta et al., 2012) and RSV (Lindquist et al., 2011). In the case of MRV, more than one eIF2α kinase is required for the induction of SGs (Qin et al., 2009). The results of our study demonstrate that inhibition of PERK, but not of PKR, HRI, or GCN2, completely abrogates SG formation and decreases the eIF2α phosphorylation level in PRRSV-infected cells, indicating that PERK is responsible for PRRSV-induced SG formation. Indeed, previous studies of other research groups had showed that PRRSV infection upregulated phosphorylation level of PERK (Huo et al., 2013) and the phosphorylation level of PKR was not well correlated with that of eIF2α in MARC-145 cells (Wang et al., 2016). Furthermore, knockdown of PKR using specific siRNA had no significant effect on PRRSV-induced eIF2α phosphorylation (Wang et al., 2016). These results suggested that PKR is not responsible for PRRSV-induced eIF2α phosphorylation and subsequent SG formation, which is consistent with our conclusion.

The endoplasmic reticulum (ER) is an important organelle structure responsible for protein synthesis (Berridge, 2002), and ER stress reduces the rates of protein translation initiation. As described above, translation arrest results in the formation of SGs (Kedersha and Anderson, 2002; Anderson and Kedersha, 2008). A previous study showed that PRRSV infection leads to an ER stress response, which, in turn, induces activation of PERK and the subsequent phosphorylation of eIF2α in MARC-145 cells (Huo et al., 2013). Thus, it is possible that there is a connection between the induction of SGs by PRRSV infection and the ER stress response.

The roles of SGs to viral replication appear to be controversial. SGs exhibit proviral or antiviral roles for different viruses. For example, expression of a cleavage-resistant G3BP1 restored SG formation during poliovirus infection and significantly inhibited virus replication (White et al., 2007). Similarly, depletion of TIA1 resulted in enhanced VSV growth and gene expression (Dinh et al., 2013). Conversely, RSV induces host SG formation to facilitate viral replication (Lindquist et al., 2010). Here, we found that, like RSV, PRRSV induces stable SG formation; however, unlike in RSV, impairment of SG formation almost had no influence on the replication of PRRSV. Similar results were previously obtained in MHV, which was reported to induce phosphorylation of eIF2α and SG assembly, but MHV replication does not benefit from the stress responses (Raaben et al., 2007).

SGs are transient and dynamic cytoplasmic RNA granules whose composition is very complex and variable. Many signaling proteins are sequestered by SGs and/or affect their assembly, which alter diverse signaling pathways (Kedersha et al., 2013). In our study, we found that the SGs are involved in the signaling pathway for the PRRSV-induced inflammatory response. In PRRSV-infected cells, impairment of SG formation by the concomitant knockdown of three SG components (TIA1, TIAR, and G3BP1) increased the mRNA levels of IL-6, IL-8, and TNF-α concomitant with an increased phosphorylation level of the NF-κB p65 subunit. Interstitial pneumonia is the most remarkable characteristic of PRRSV infection, suggesting that the inflammatory response plays an important role in the infection and pathogenesis induced by PRRSV. Inflammation is also a two-edged sword for the host. Impairment of SG assembly enhanced the expression of PRRSV-induced inflammatory cytokines to some extent, indicating that SGs may be a host defense factor against the PRRSV-induced inflammatory response, even though it is not involved in PRRSV replication.

A limitation of this study is that only MARC-145 cells were used to study the formation of SGs during PRRSV infection. MARC-145 cells are not natural host cells for PRRSV, it is therefore vital to detect the relationship between PRRSV and SGs in primary porcine alveolar macrophages (PAMs) in future study. In addition, a genotype 2 PRRSV strain WUH3 was used in our study. Previous studies have showed that cytokine expression profiles of PRRSV-infected cells vary with different genotype isolates (Lee and Lee, 2012; Chen, 2015). Whether SG assembly is associated with the expression of genotype 1 PRRSV-induced inflammatory cytokines needs further study.

In summary, our present study demonstrates that PRRSV infection induces the stable formation of SGs through a PERK-dependent mechanism. Impairment of SG assembly does not significantly affect PRRSV replication; however, the impairment does enhance the PRRSV-induced inflammatory response. SG formation and the inflammatory response during PRRSV infection may be connected with ER stress, and elucidating this issue will help us reach a better understanding of the pathogenesis and immune regulation mechanism(s) of PRRSV.

## Author contributions

YZ and SX designed research; YZ, KC, and DW performed research; YZ and LF analyzed data; YZ, HC, and SX wrote the paper.

## Funding

This work was supported by the National Natural Sciences Foundation of China (31490602, 31372467), the National Basic Research Program (973) of China (2014CB542700), and the Natural Science Foundation of Hubei Province (2014CFA009).

### Conflict of interest statement

The authors declare that the research was conducted in the absence of any commercial or financial relationships that could be construed as a potential conflict of interest.
